# Supplementary material for: Uncovering hidden enhancers through unbiased in vivo testing
Source: Nat Commun. 2025 Aug 8;16:7313. doi: 10.1038/s41467-025-62497-0 (PMC12331988; doi:10.1038/s41467-025-62497-0)
Supplement: Supplementary file 1 — Supplementary Information [file 41467_2025_62497_MOESM1_ESM.pdf]

SUPPLEMENTARY INFORMATION

Uncovering Hidden Enhancers Through Unbiased *In Vivo* Testing

Brandon J. Mannion[1,2], Stella Tran[1], Ingrid Plajzer-Frick[1], Catherine S. Novak[1], Veena Afzal[1], Jennifer A. Akiyama[1], Ismael Sospedra-Arrufat[3], Sarah Barton[1], Erik Beckman[1], Tyler H. Garvin[1], Patrick Godfrey[1], Janeth Godoy[1], Riana D. Hunter[1], Momoe Kato[1], Michael Kosicki[1], Anne N. Kronshage[1], Elizabeth A. Lee[1], Eman M. Meky[1], Quan T. Pham[1], Kianna von Maydell[1], Yiwen Zhu[1], Javier Lopez-Rios[3,4], Diane E. Dickel[1,9], Marco Osterwalder[5,6,*], Axel Visel[1,7,8,*] and Len A. Pennacchio[1,2,7,*].

[1] Environmental Genomics & System Biology Division, Lawrence Berkeley National Laboratory, 1 Cyclotron Road, Berkeley, CA 94720, USA

[2] Comparative Biochemistry Program, University of California, Berkeley, CA 94720, USA

[3] Centro Andaluz de Biología del Desarrollo, Consejo Superior de Investigaciones Científicas, Universidad Pablo de Olavide, and Junta de Andalucía, Sevilla 41013, Spain

[4] Universidad Loyola Andalucía, School of Health Sciences, Seville Campus, 41704, Dos Hermanas, Seville, Spain

[5] Department for BioMedical Research (DBMR), University of Bern, Bern, Switzerland.

[6] Department of Cardiology, Bern University Hospital, Bern, Switzerland.

[7] U.S. Department of Energy Joint Genome Institute, 1 Cyclotron Road, Berkeley, CA 94720, USA

[8] School of Natural Sciences, University of California, Merced, Merced, California, USA

[9] Present address: Octant, Inc.; Emeryville, CA, USA

* To whom correspondence should be addressed: M.O., marco.osterwalder@unibe.ch; A.V., AVisel@lbl.gov; L.A.P., LAPennacchio@lbl.gov

Supplementary Figures

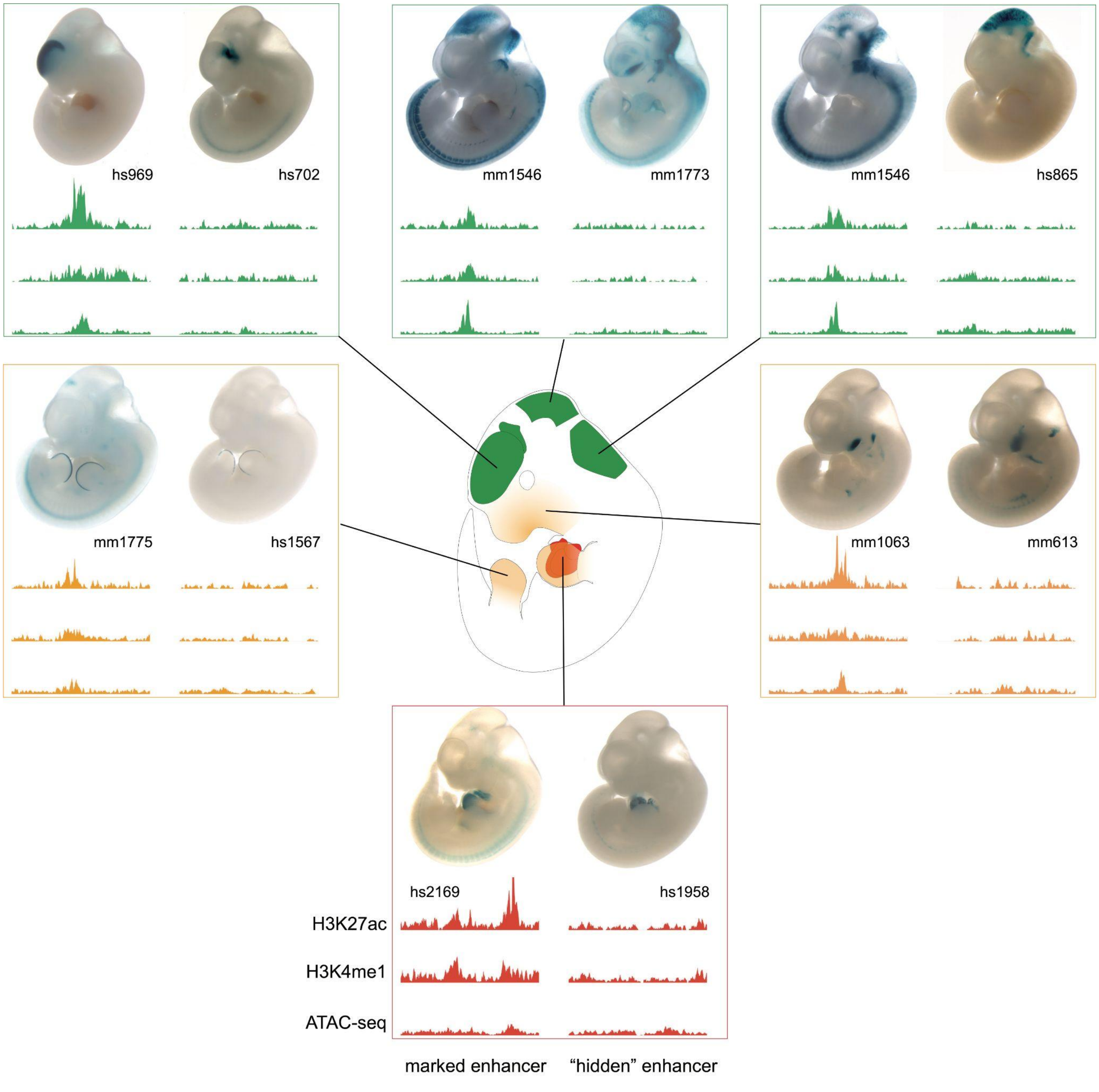

Supplementary Figure 1. Mouse *in vivo* enhancers with and without canonical enhancer-associated chromatin marks. Representative transgenic result (mouse E11.5 embryos) displayed above tissue-specific chromatin profile for each tested element (VISTA ID provided). For each of the 6 considered tissues, an active enhancer with canonical enhancer-associated chromatin marks (left) is displayed alongside an active enhancer without canonical enhancer-associated chromatin marks (right). Mouse tissue- and stage-matched H3K27ac ChIP-seq, H3K4me1 ChIP-seq, ATAC-seq are from ENCODE[1].

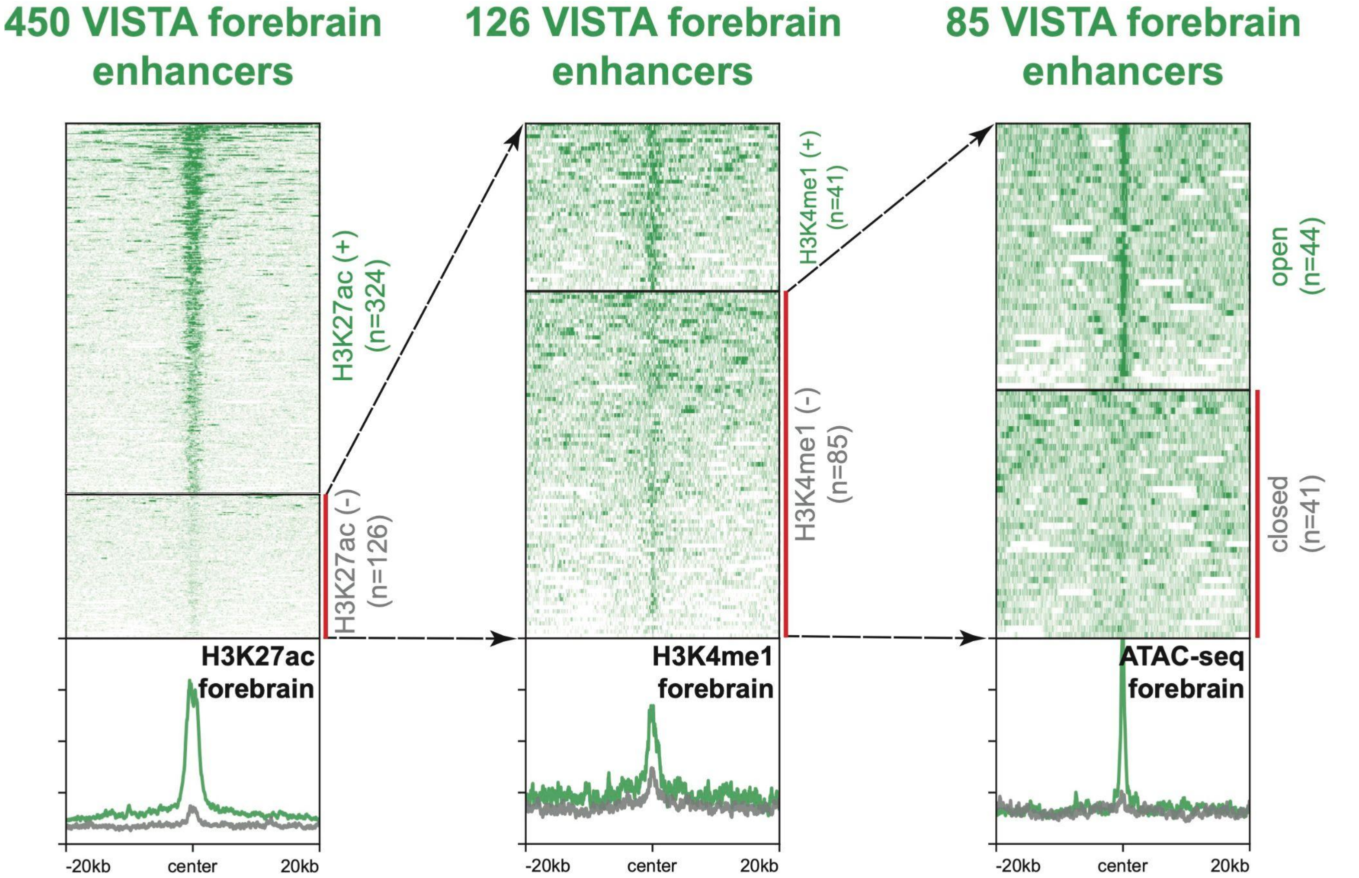

Supplementary Figure 2. Chromatin profiles of active forebrain enhancers with and without H3K27ac, H3K4me1, and ATAC-seq (open chromatin). Forebrain enhancers from the VISTA Enhancer browser stratified across three canonical enhancer-associated chromatin marks. Processed mouse chromatin data are from ENCODE[1].

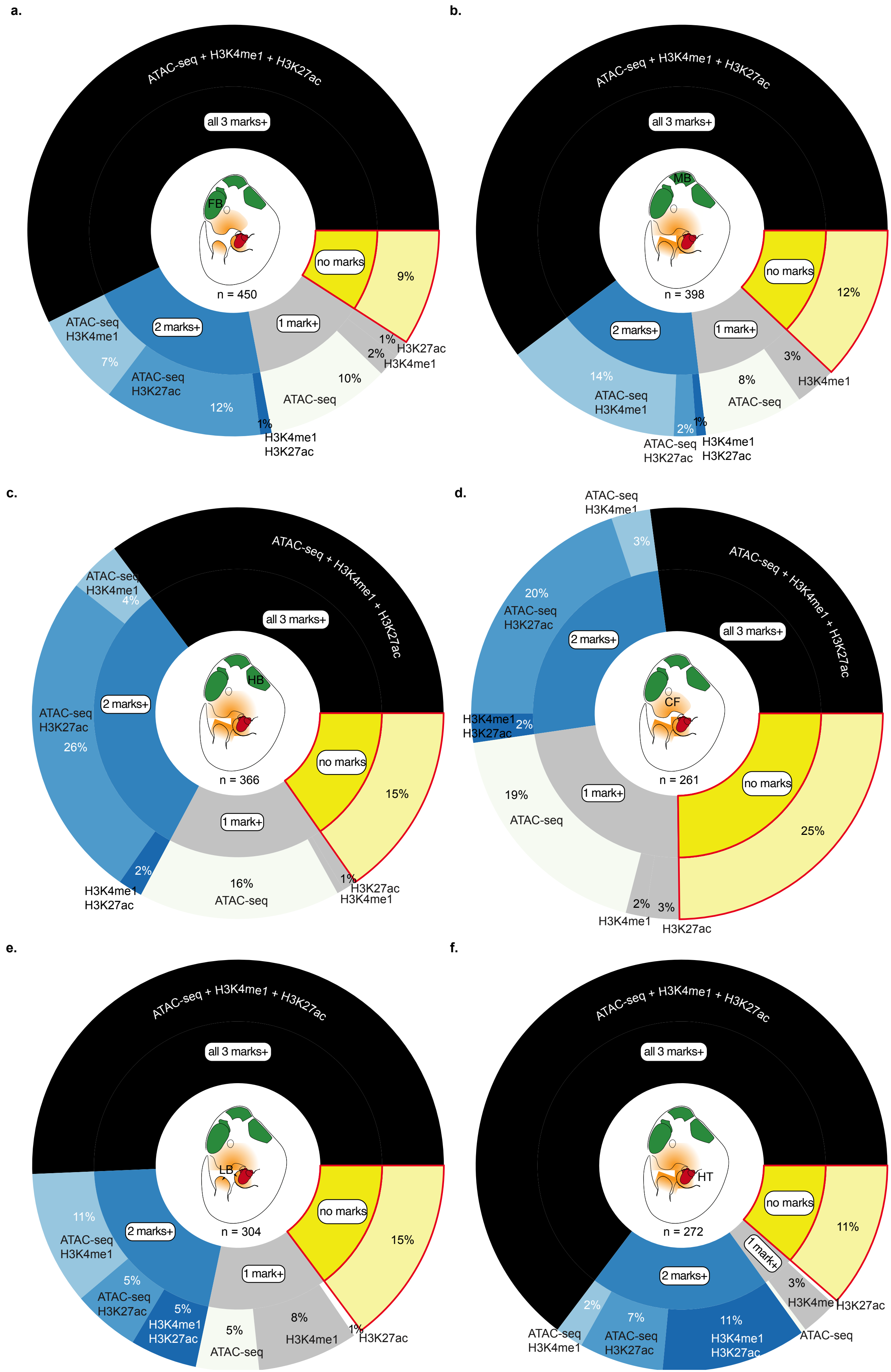
a.
ATAC-seq + H3K4me1 + H3K27ac
all 3 marks+
FB
n = 450
no marks
9%
2 marks+
1 mark+
ATAC-seq
H3K4me1
7%
ATAC-seq
H3K27ac
12%
1%
H3K4me1
H3K27ac
10%
ATAC-seq
1%
H3K27ac
2%
H3K4me1
b.
ATAC-seq + H3K4me1 + H3K27ac
all 3 marks+
MB
n = 398
no marks
12%
2 marks+
1 mark+
3%
H3K4me1
14%
ATAC-seq
H3K4me1
8%
ATAC-seq
2%
ATAC-seq
H3K27ac
1%
H3K4me1
H3K27ac
c.
ATAC-seq + H3K4me1 + H3K27ac
all 3 marks+
ATAC-seq
H3K4me1
4%
2 marks+
ATAC-seq
H3K27ac
26%
HB
n = 366
no marks
15%
1 mark+
2%
H3K4me1
H3K27ac
16%
ATAC-seq
1%
H3K27ac
H3K4me1
d.
ATAC-seq
H3K4me1
3%
ATAC-seq + H3K4me1 + H3K27ac
20%
ATAC-seq
H3K27ac
2 marks+
all 3 marks+
H3K4me1
H3K27ac
2%
CF
n = 261
1 mark+
no marks
19%
ATAC-seq
25%
2%
3%
H3K4me1
H3K27ac
e.
ATAC-seq + H3K4me1 + H3K27ac
all 3 marks+
LB
n = 304
no marks
15%
2 marks+
11%
ATAC-seq
H3K4me1
1 mark+
5%
ATAC-seq
H3K27ac
5%
H3K4me1
H3K27ac
5%
ATAC-seq
8%
H3K4me1
1%
H3K27ac
f.
ATAC-seq + H3K4me1 + H3K27ac
all 3 marks+
HT
n = 272
no marks
11%
1 mark+
2 marks+
3%
H3K4me
H3K27ac
ATAC-seq
2%
ATAC-seq
H3K4me1
7%
ATAC-seq
H3K27ac
11%
H3K4me1
H3K27ac

Supplementary Figure 3. Proportions of VISTA enhancers with enhancer-associated chromatin signatures by tissue. Active enhancers across the six considered tissues with different combinations of canonical enhancer-associated chromatin marks. For every case there are active enhancers that do not have any of these considered marks. The tissues/regions are: **(a)** forebrain, **(b)** midbrain, **(c)** hindbrain, **(d)** craniofacial, **(e)** limb, and **(f)** heart.

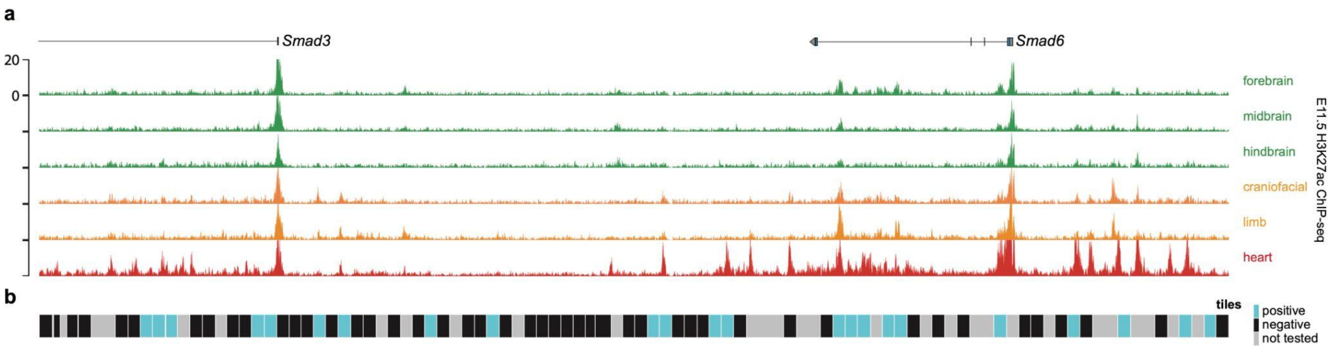

Supplementary Figure 4. Tiling a second locus for the unbiased identification of mouse in vivo enhancers. (a) *Smad3*/*Smad6* locus with mouse E11.5 H3K27ac ChIP-seq data (ENCODE) for six tissues. **(b)** Elements (~5kb in size and overlapping with adjacent elements) designed for the unbiased tiling assay. Elements that were tested and that had reproducible enhancer-reporter activity (in one or more tissues) in the mouse *in vivo* transgenic assay are shaded blue. Elements not tested are shaded gray.

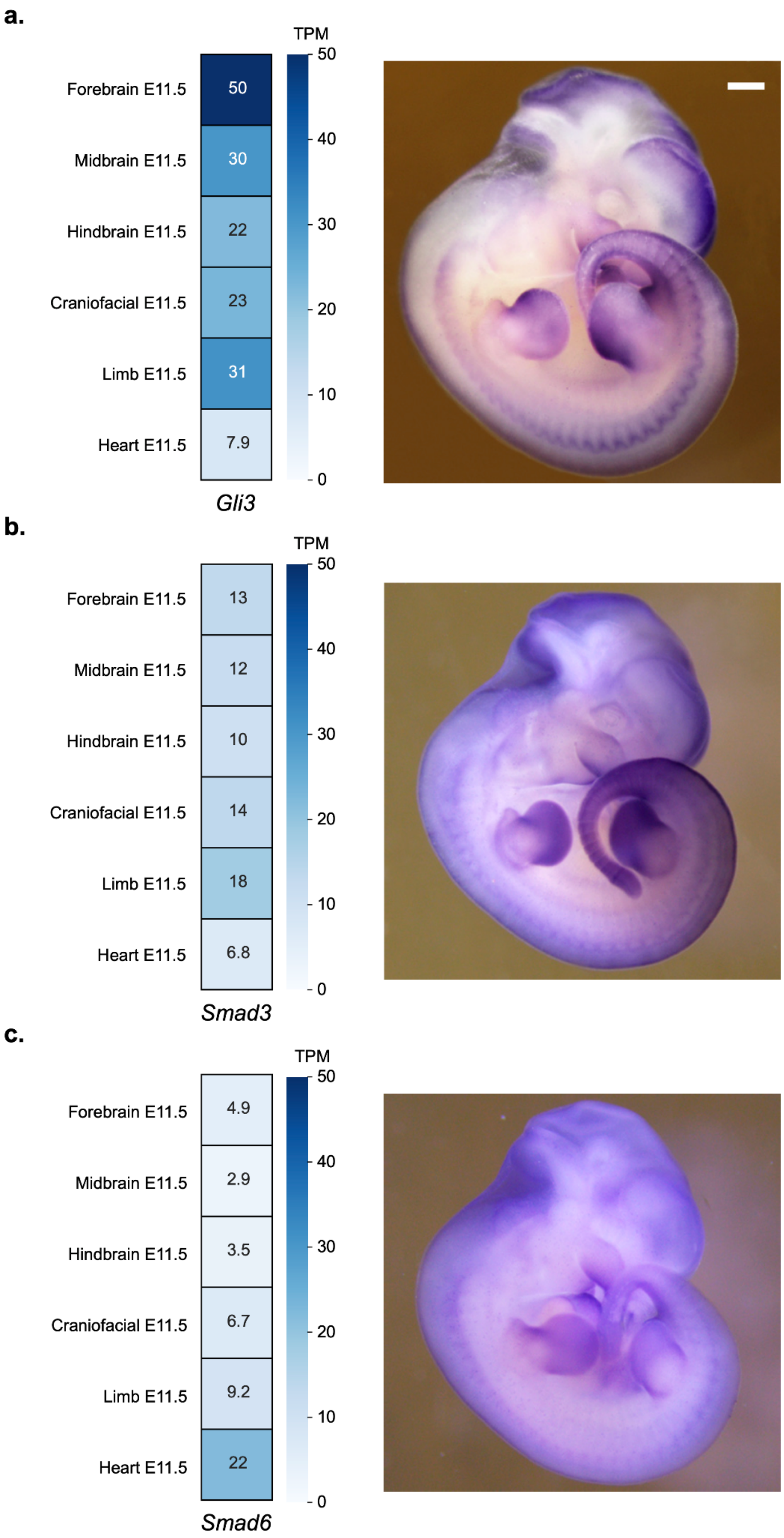

Supplementary Figure 5. Mouse E11.5 gene expression by tissue for the *Gli3*, *Smad3*, and *Smad6* genes. (a) Per tissue RNA-seq and mouse *in situ* data for (**a)** *Gli3*, **(b)** *Smad3,* and **(c)** *Smad6* genes. RNA-seq data are from mouse ENCODE[2]. TPM, transcripts per million.

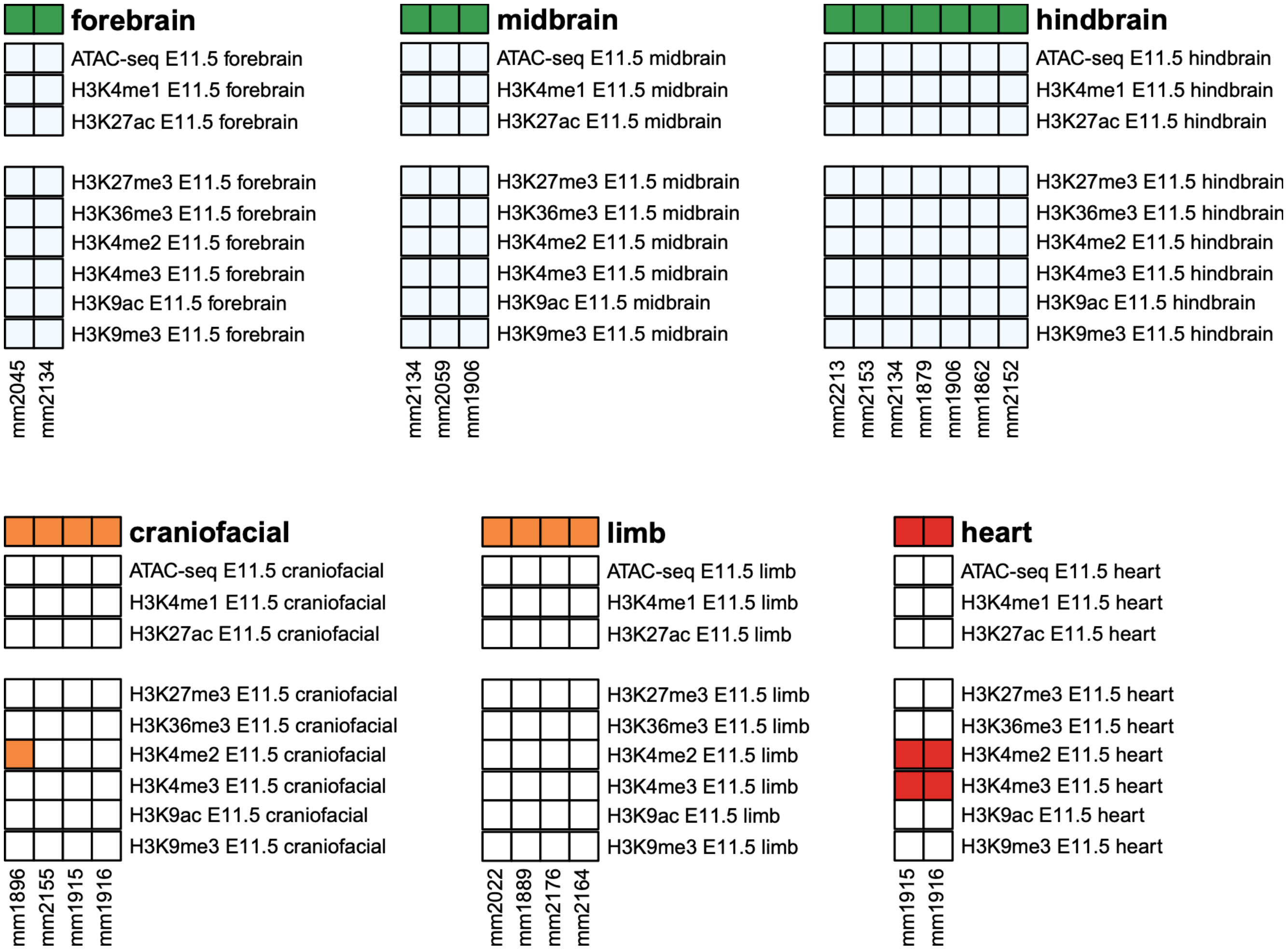

Supplementary Figure 6. Hidden enhancers commonly lack other chromatin marks at their endogenous site. A majority of hidden enhancers identified from the unbiased tiling (across the *Gli3* and *Smad3*/*Smad6* loci) do not have other chromatin marks. Processed mouse chromatin data are from ENCODE[1].

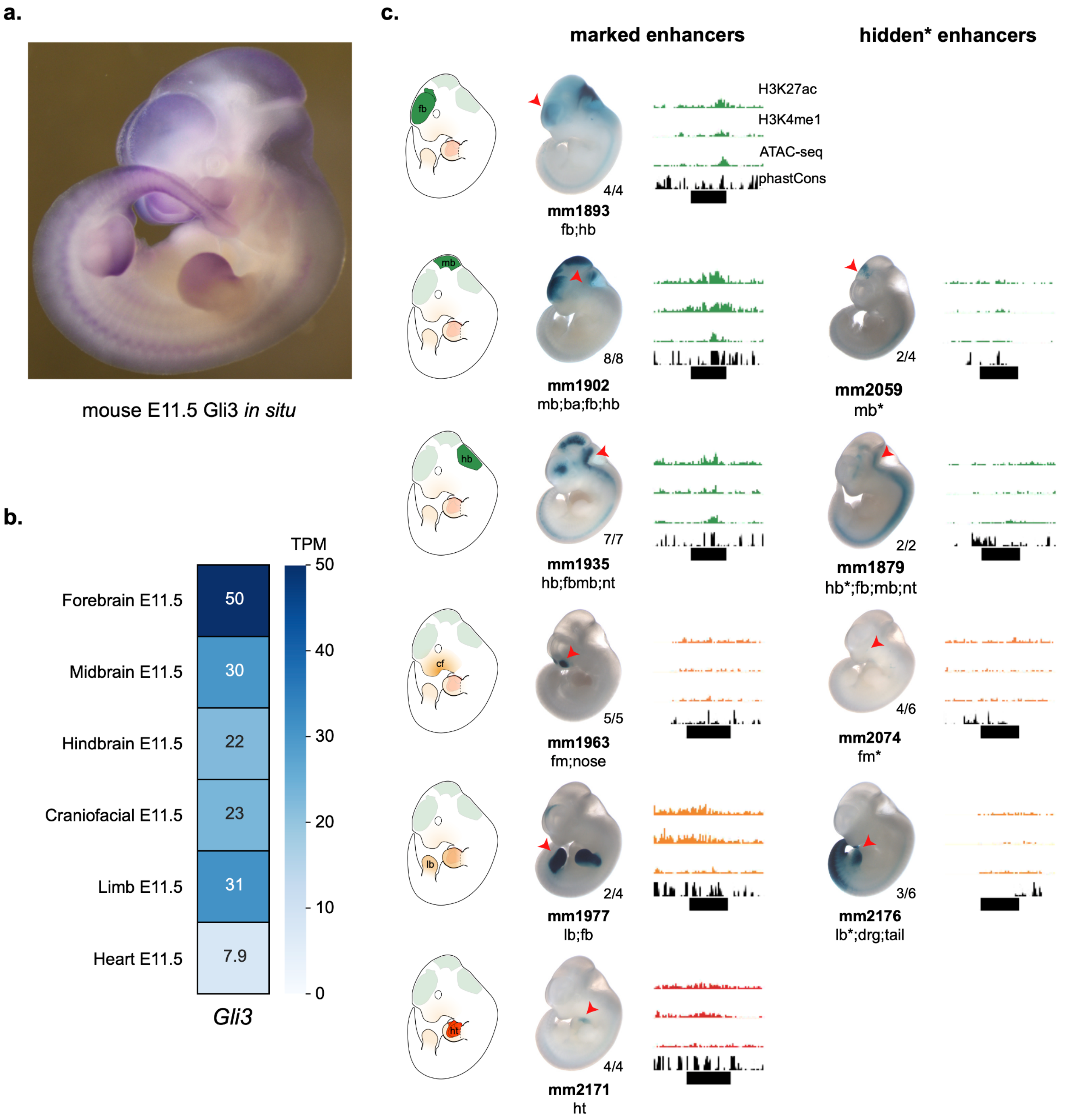

Supplementary Figure 7. Hidden enhancers within the *Gli3* locus show similar tissue-specific reporter activities as their marked counterparts and correlate with *Gli3 in situ* expression data. **(a)** Whole-mount *in situ* for *Gli3* in E11.5 mouse. **(b)** ENCODE RNA-seq data from E11.5 mouse across six developmental tissues. TPM, transcripts per million. **(c)** Examples of marked and hidden enhancers identified across the *Gli3* locus*.* Red ticks mark regions with reproducible tissue-specific enhancer reporter activity, summarized by one example transgenic embryo. Indicated with each representative transgenic result is the number of independent embryos with LacZ staining in the considered tissue over the total number of transgenic embryos obtained. Black bars underneath the chromatin and evolutionary conservation tracks represent the candidate element that was tested in the mouse *in vivo* transgenic reporter assay.

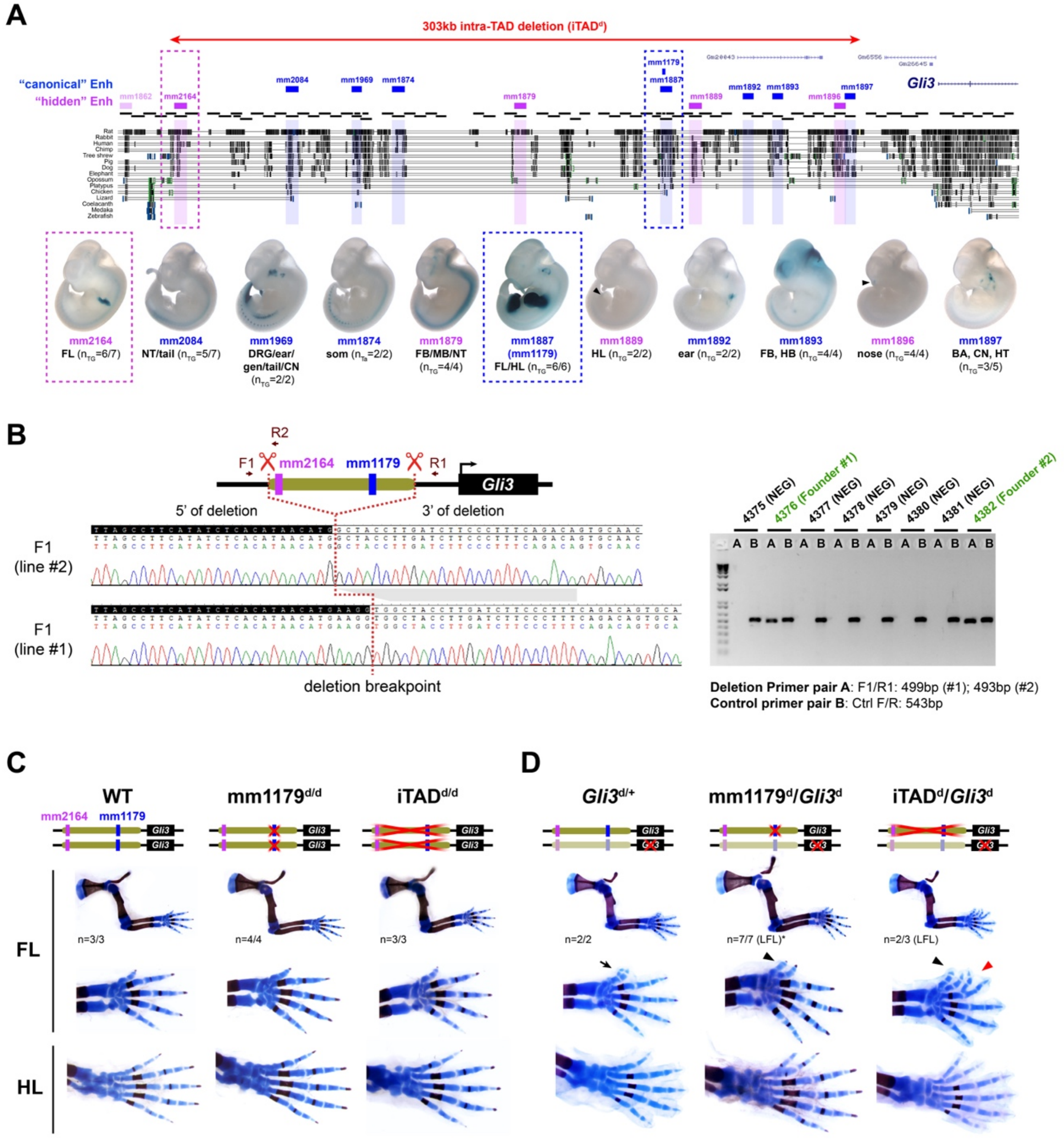

Supplementary Figure 8. Assessment of hidden enhancer function in embryonic limb development. (**A**) Deletion of a 303kb-spanning *Gli3* upstream intra-TAD genomic interval (iTAD^{del}, red double arrow) containing seven "canonical" (blue) and four "hidden" (magenta) enhancers as identified using enSERT transgenic reporter analysis of tiling elements across the *Gli3* locus (**Figure 2**). The mm1887/mm1179 canonical enhancer region described previously[3,4] and the hidden enhancer mm2164 (both indicated by dashed lines) represent the only elements with strong and highly reproducible activity in the anterior mesenchyme of the developing limb. Vista Enhancer Browser IDs are indicated (mm, *Mus musculus*). UCSC browser Multiz Alignment of 60 vertebrates is shown, with tiled elements assessed by reporter transgenesis indicated above. FL/HL: fore- and hindlimb. NT, neural tube. DRG, dorsal root ganglia. Gen, genitalia. CN, cranial nerve. Som, somites. FB/MB/HB: fore-, mid and hindbrain. BA, branchial arch. HT, heart. (**B**) Left: Two nearly identical iTAD^{del} mouse lines (#1 and #2) were established using CRISPR/Cas9 from founders with clean deletion breakpoints, as determined by genotyping PCR and Sanger sequencing (Supplementary Tables 1 and 2) and as performed previously

for large genomic regions[5]. (**C, D**) Comparative skeletal analysis of fore- and hindlimbs of mouse embryos at E18.5. Neither homozygous deletion of the mm1179 enhancer[4] nor the iTAD region (combined mm1179/mm2164 limb enhancer deletion) leads to visible limb phenotypes (**C**). While *Gli3* heterozygous ($\mathit{Gli3}^{d/+)}$ embryos display a split digit 1 (arrow), removal of mm1179 in *Gli3* dosage-reduced conditions ($mm1179^{d}/\mathit{Gli3}^{d}$) results in digit 1 duplication consistently in the LFL (black arrowhead)[4] (**D**). In contrast, removal of both the “canonical” mm1179 and the “hidden” mm2164 limb enhancers via deletion of the iTAD region in *Gli3* dosage-reduced conditions ($iTAD^{d}/\mathit{Gli3}^{d}$) results in an exacerbated LFL phenotype including duplicated digit 1 and additional bifurcation of digit 2 (red arrowhead). Images from $mm1179^{d/d}$ limb skeletons are derived from our previous study[4]. Circled boxes mark enhancers with activity in the limb.“ n” denotes number of biological replicates with similar results.

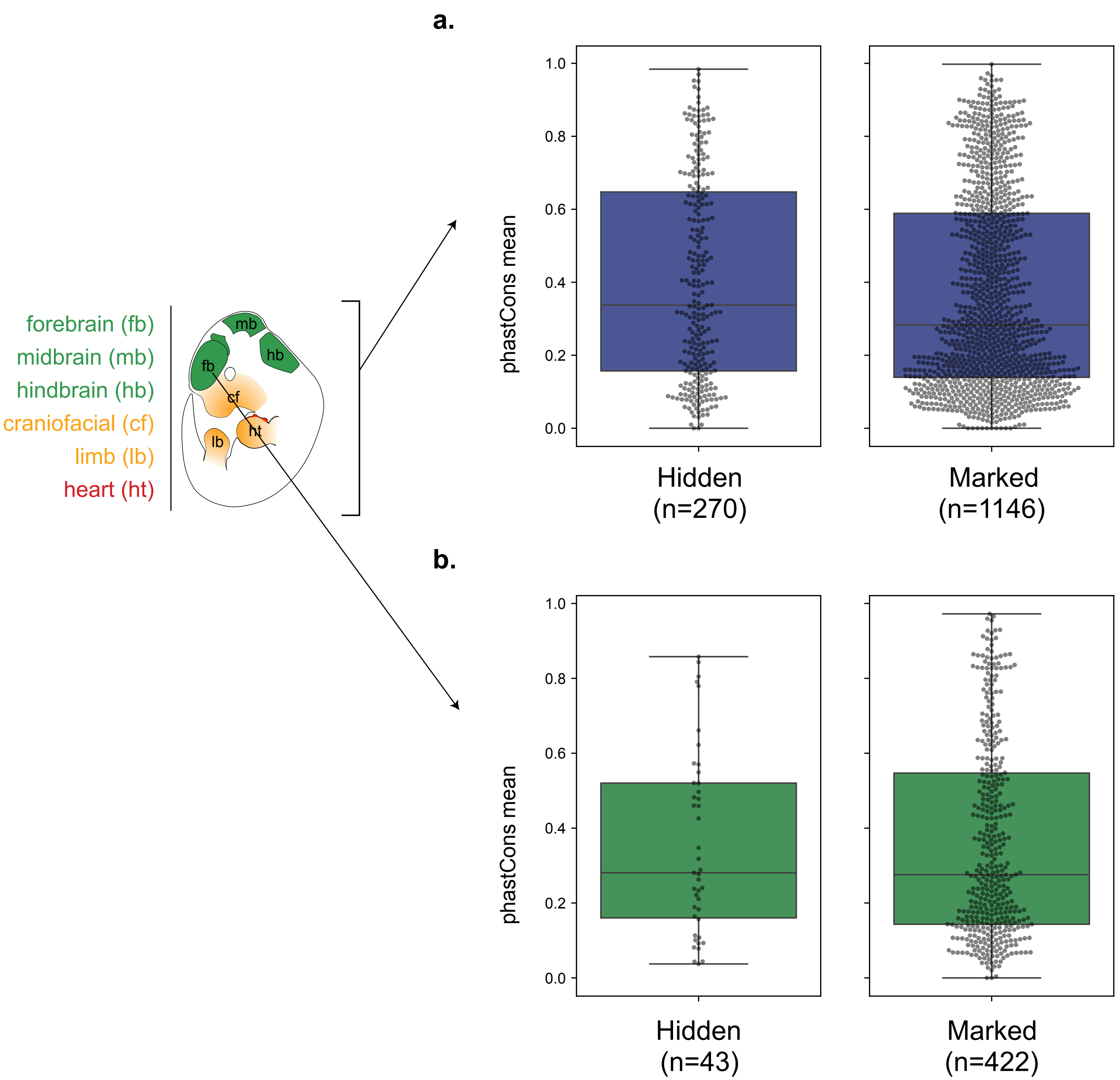

Supplementary Figure 9. Similar levels of evolutionary conservation (phastCons) between hidden enhancers and marked enhancers. Hidden enhancers and marked enhancers have similar levels of evolutionary conservation (phastCons) for **(a)** all tissues considered together and also for each considered tissue, exemplified by **(b)** forebrain enhancers. No statistically significant difference via Kolmogorov-Smirnov comparison. Box plots indicate interquartile range, median, maximum/minimum values (bars). Dots represent individual data points. Source data are provided as a Source Data file.

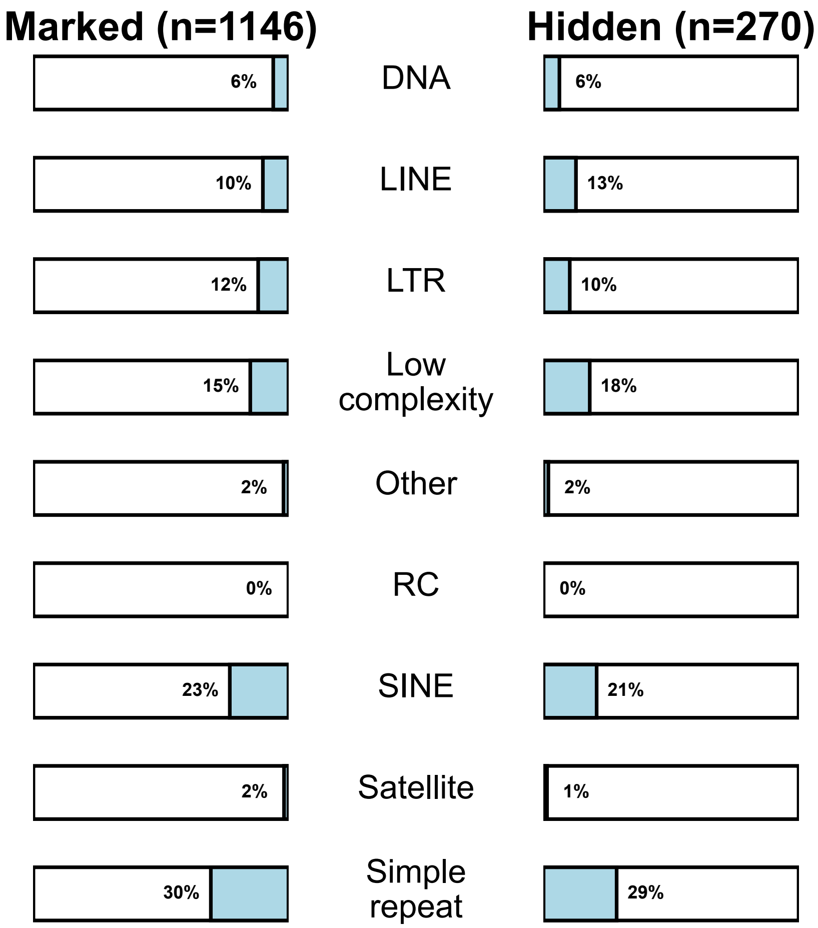

Supplementary Figure 10. Similar proportions of transposable element families between marked and hidden enhancers. The proportions of transposable element classes among positive elements are comparable between marked and hidden enhancers (across all six tissues). All elements were evaluated for their repeat content from RepeatMasker mouse mm10 annotations.

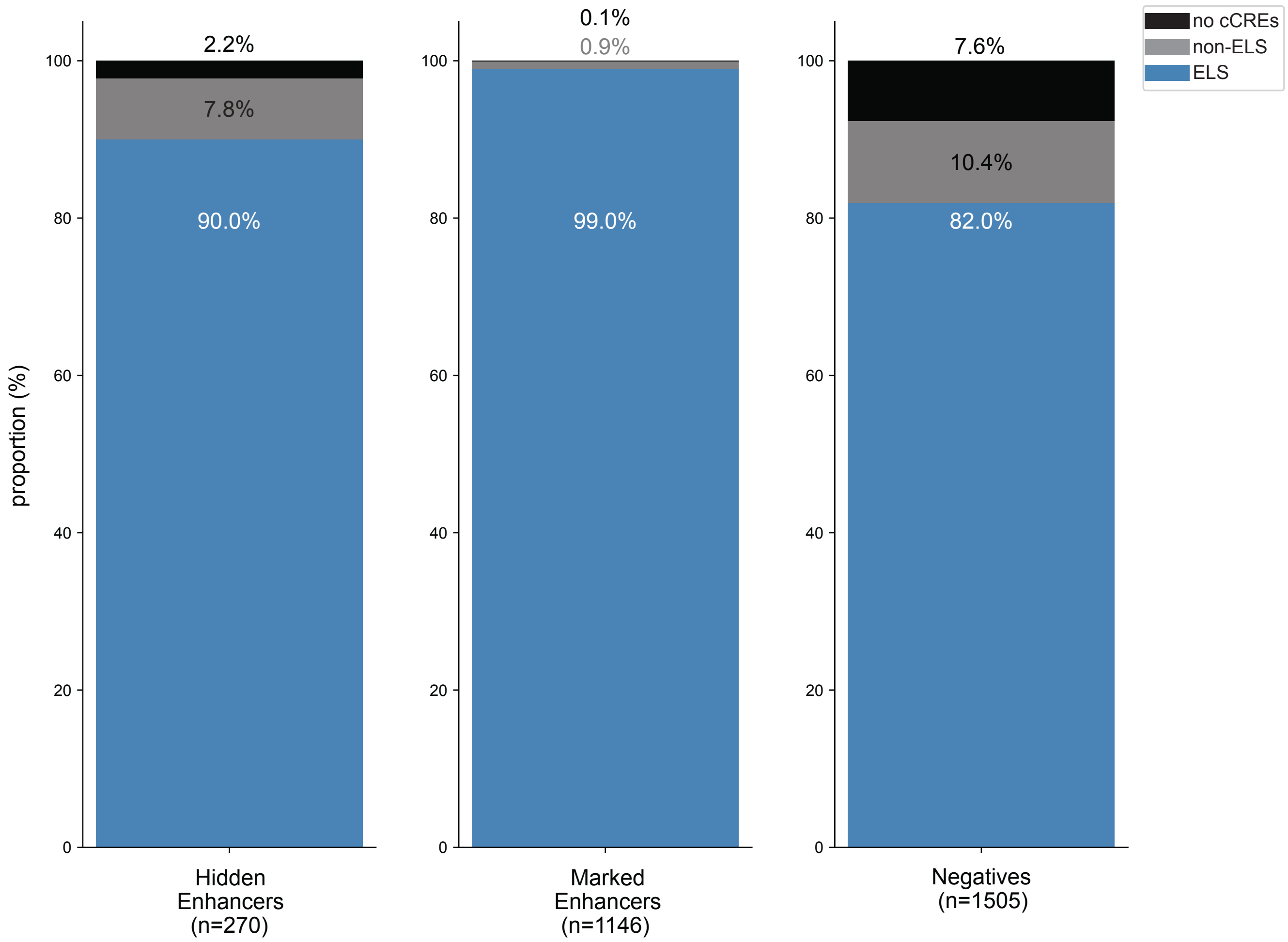

Supplementary Figure 11. Majority of hidden enhancers identified from the retrospective VISTA and unbiased tiling studies contain candidate cis-regulatory elements (cCREs) that are derived from multiple tissue types and developmental stages. cCREs with enhancer-like signatures (ELS[6]) are present in the hidden enhancers identified across the retrospective VISTA and tiling studies. ELS cCREs are also present in a majority of active enhancers with canonical enhancer-associated chromatin marks (marked enhancers). A majority of negative elements (did not show reproducible tissue-specific enhancer-reporter activity at E11.5) also overlap with ELS cCREs. Source data are provided as a Source Data file.

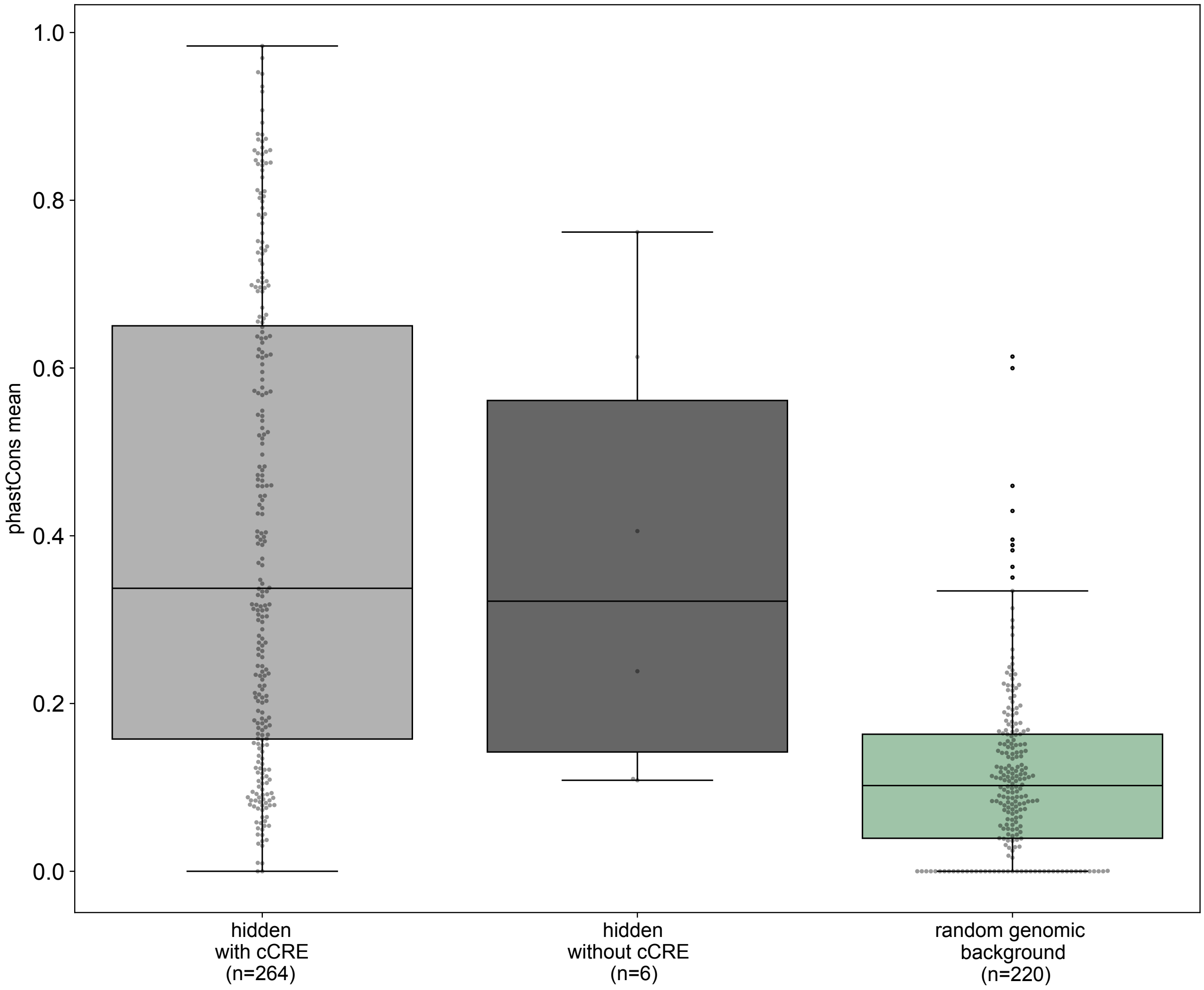

Supplementary Figure 12. Hidden enhancers that do not overlap with candidate cis-regulatory elements (cCREs) have similar levels of evolutionary conservation (phastCons) as those that do. Among hidden enhancers identified across the retrospective VISTA and tiling studies, those that do not overlap with cCREs[6] have similar levels of elevated evolutionary conservation (compared to random genomic background) as those that do. Box plots indicate interquartile range, median, maximum/minimum values (bars). Dots represent individual data points. Source data are provided as a Source Data file.

Supplementary Tables

Supplementary Table 1. Overview of VISTA E11.5 enhancers by tissue. Note: Enhancers active in branchial arch, facial mesenchyme, and/or nose are grouped as craniofacial enhancers. Enhancers analyzed in this study are marked gray.

Tissue	Total elements
forebrain	450
midbrain	398
hindbrain	366
neural tube	256
dorsal root ganglion	84
somite	58
cranial nerve	62
trigeminal V	56
facial mesenchyme	95
branchial arch	164
nose	85
ear	29
eye	90
limb	304
heart	272
liver	8
blood vessels	24
tail	32
genital tubercle	12

Supplementary Table 2. Single guide (sg) RNA sequences used for CRISPR engineering of the *Gli3* iTAD^{del} allele in mouse embryos. The use of unique 5' and 3'-targeted sgRNA sequences resulted in the generation of two nearly identical founder lines (see Supplementary Fig. 8B and Methods).

Mouse allele	Deleted region genomic coordinates (mm10)	Deleted region length (bp)	5' sgRNA target sequence (5'-3')	3' sgRNA target sequence (5'-3')
***Gli3* iTAD^{del}** (Line #1)	chr13:15126903-15429781	302879	CCTCAGTGGAGGATCTTGAAGCC	CTCATGCTGCTTACCACAGTGG
***Gli3* iTAD^{del}** (Line #2)	chr13:15126899-15429783	302885	CCTCAGTGGAGGATCTTGAAGCC	CTCATGCTGCTTACCACAGTGG

Supplementary Table 3. Primers used for screening and genotyping of *Gli3* iTAD^{del} mice. PCR genotyping strategy and results using agarose gel electrophoresis are displayed in Supplementary Fig. 8B. N.a., not amplified. Del, deletion. #, mouse line.

Analyzed Region	Primer ID	Sequence	Product size
***Gli3* iTAD**	**F1**	acgttccaggcctaggtaatatc	WT: 303378bp (n.a.)
(deletion)	**R1**	tgcctatagtattcccctgatga	iTAD^{del}: 499bp (#1), 493bp (#2)
***Gli3* iTAD**	**F1**	acgttccaggcctaggtaatatc	WT: 887bp
(5’ junction)	**R2**	tagagatgcacatcaactcctca	iTAD^{del}: n.a.
**Control**	**F**	agctggtagccttaaaataagccaa	WT/ iTAD^{del}: 543bp
(*Gli3* intronic)	**R**	gcctgaaagaggtcatcatcacc	

Supplementary Table 4. Summary of hidden enhancer transcription factor motif analysis.
Parameters: -size given -len 8,9,10,12,14 with background = all positive VISTA enhancers.

Tissue	Motif Name	Consensus	Result
forebrain	n/a	n/a	n/a
midbrain	n/a	n/a	n/a
hindbrain	NeuroD1(bHLH)/Islet-NeuroD1-ChIP-Seq(GSE30298)/Homer	GCCATCTGTT	*not significant*
hindbrain	HOXA2(Homeobox)/mES-Hoxa2-ChIP-Seq(Donaldson_et_al.)/Homer	GYCATCMATCAT	*not significant*
craniofacial	n/a	n/a	n/a
limb	Foxh1(Forkhead)/hESC-FOXH1-ChIP-Seq(GSE29422)/Homer	NNTGTGGATTSS	*not significant*
limb	GABPA(ETS)/Jurkat-GABPa-ChIP-Seq(GSE17954)/Homer	RACCGGAAGT	*not significant*
limb	ETS:RUNX(ETS,Runt)/Jurkat-RUNX1-ChIP-Seq(GSE17954)/Homer	RCAGGATGTGGT	*not significant*
heart	n/a	n/a	n/a
all tissue	n/a	n/a	n/a

Supplementary Table 5. Summary of hidden enhancer functional enrichment analysis.
Parameters: a) basal plus extension (5000bp upstream, 1000bp downstream, distal up to 1Mbp)

**Tissue**	**Result** (hidden enhancer region vs. background = hidden + marked enhancer regions)
forebrain	n/a
midbrain	n/a
hindbrain	n/a
craniofacial	n/a
limb	n/a
heart	5 GO Biological Processes that meet FDR threshold: melanocyte differentiation; developmental pigmentation; pigment cell differentiation; pigmentation; positive regulation of gliogenesis
all hidden enhancers	n/a

References

1. Gorkin, D. U. *et al.* An atlas of dynamic chromatin landscapes in mouse fetal development. *Nature* **583**, 744–751 (2020).

2. He, P. *et al.* The changing mouse embryo transcriptome at whole tissue and single-cell resolution. *Nature* **583**, 760–767 (2020).

3. Osterwalder, M. *et al.* HAND2 targets define a network of transcriptional regulators that compartmentalize the early limb bud mesenchyme. *Dev. Cell* **31**, 345–357 (2014).

4. Osterwalder, M. *et al.* Enhancer redundancy provides phenotypic robustness in mammalian development. *Nature* **554**, 239–243 (2018).

5. Abassah-Oppong, S. *et al.* A gene desert required for regulatory control of pleiotropic Shox2 expression and embryonic survival. *Nat. Commun.* **15**, 8793 (2024).

6. ENCODE Project Consortium *et al.* Expanded encyclopaedias of DNA elements in the human and mouse genomes. *Nature* **583**, 699–710 (2020).
