# Supplementary material for: Uncovering hidden enhancers through unbiased in vivo testing
Source: Nat Commun. 2025 Aug 8;16:7313. doi: 10.1038/s41467-025-62497-0 (PMC12331988; doi:10.1038/s41467-025-62497-0)
Supplement: Supplementary file 2 — Description of Additional Supplementary Files [file 41467_2025_62497_MOESM2_ESM.pdf]

Description of Additional Supplementary Files

Supplementary Data 1. ENCODE mouse chromatin and RNA-seq data.

Supplementary Data 2. Chromatin intersections of enhancers from the VISTA retrospective study and the unbiased tiling.

Supplementary Data 3. Tissue-specific H3K27ac peak counts across the two loci tested by tiling for enhancer activity.
