# Supplementary material for: Uncovering hidden enhancers through unbiased in vivo testing
Source: Nat Commun. 2025 Aug 8;16:7313. doi: 10.1038/s41467-025-62497-0 (PMC12331988; doi:10.1038/s41467-025-62497-0)
Supplement: Supplementary file 6 — Reporting Summary [file 41467_2025_62497_MOESM6_ESM.pdf]

nature portfolio

Corresponding author(s): Len Pennacchio, Axel Visel, Marco Osterwalder

Last updated by author(s): Dec 27, 2024

Reporting Summary

Nature Portfolio wishes to improve the reproducibility of the work that we publish. This form provides structure for consistency and transparency in reporting. For further information on Nature Portfolio policies, see our Editorial Policies and the Editorial Policy Checklist.

Statistics

For all statistical analyses, confirm that the following items are present in the figure legend, table legend, main text, or Methods section.

n/a	Confirmed	
☐	☒	The exact sample size (*n*) for each experimental group/condition, given as a discrete number and unit of measurement
☒	☐	A statement on whether measurements were taken from distinct samples or whether the same sample was measured repeatedly
☐	☒	The statistical test(s) used AND whether they are one- or two-sided<br>*Only common tests should be described solely by name; describe more complex techniques in the Methods section.*
☒	☐	A description of all covariates tested
☒	☐	A description of any assumptions or corrections, such as tests of normality and adjustment for multiple comparisons
☐	☒	A full description of the statistical parameters including central tendency (e.g. means) or other basic estimates (e.g. regression coefficient) AND variation (e.g. standard deviation) or associated estimates of uncertainty (e.g. confidence intervals)
☐	☒	For null hypothesis testing, the test statistic (e.g. *F*, *t*, *r*) with confidence intervals, effect sizes, degrees of freedom and *P* value noted<br>*Give P values as exact values whenever suitable.*
☒	☐	For Bayesian analysis, information on the choice of priors and Markov chain Monte Carlo settings
☒	☐	For hierarchical and complex designs, identification of the appropriate level for tests and full reporting of outcomes
☒	☐	Estimates of effect sizes (e.g. Cohen's *d*, Pearson's *r*), indicating how they were calculated

Our web collection on statistics for biologists contains articles on many of the points above.

Software and code

Policy information about availability of computer code

Data collection	No commercial, open source or custom software or code were used for data collection in this study.
Data analysis	Processed mouse chromatin data (ATAC-seq; ChIP-seq for H3K27ac, H3K4me1, H3K4me2, H3K4me3, H3K27me3, H3K9ac, H3K36me3, H3K9me3) and RNA-seq data were downloaded from the ENCODE resource portal (https://www.encodeproject.org/). Details on the generation and processing of these data are available here: https://www.encodeproject.org/pipelines/. See Table S1 for a listing of all the bulk tissue mouse data used for chromatin intersections or tissue expression analyses.<br>Mouse in vivo validated elements from both the VISTA Enhancer Browser and the tiling assay were intersected with tissue-specific ENCODE chromatin data via bedtools (v2.29.0) to check for the presence or absence of enhancer-associated chromatin signatures (e.g., tissue-specific mouse E11.5 peaks from H3K27ac ChIP-seq, H3K4me1 ChIP-seq, and/or ATAC-seq data) within each elements' genomic coordinates. Elements with reproducible enhancer-reporter activity (positive elements) but without any of the three enhancer-associated chromatin signatures in the relevant tissue(s) were designated as hidden enhancers. Positive elements with any (up to all) of the three enhancer-associated chromatin signatures were considered marked enhancers. Positive elements were also checked for overlap with other chromatin features available from mouse ENCODE: DNase-seq, H3K27me3 ChIP-seq, H3K36me3 ChIP-seq, H3K4me2 ChIP-seq, H3K4me3 ChIP-seq, H3K9ac ChIP-seq, and H3K9me3 ChIP-seq. Both mouse embryonic days 10.5 (E10.5) and 11.5 (E11.5) data were used for the above analyses. HOMER (v4.10) was used for transcription factor motif analysis and GREAT (v4.0.4) was used for functional enrichment analysis.

For manuscripts utilizing custom algorithms or software that are central to the research but not yet described in published literature, software must be made available to editors and reviewers. We strongly encourage code deposition in a community repository (e.g. GitHub). See the Nature Portfolio guidelines for submitting code & software for further information.

Data

Policy information about availability of data

All manuscripts must include a data availability statement. This statement should provide the following information, where applicable:

- Accession codes, unique identifiers, or web links for publicly available datasets
- A description of any restrictions on data availability
- For clinical datasets or third party data, please ensure that the statement adheres to our policy

Accession codes of previously published ATAC-seq and ChIP-seq datasets reprocessed in this study are listed in Supplementary Table S1. Wherever applicable, reference genomes Mouse GRCm38/mm10 and Human GRCh38/hg38 were used for alignment and comparisons. Images of transgenic embryos with Lacz-reporter activity are available at the Vista Enhancer Browser (http://enhancer.lbl.gov). Correspondence and requests for materials should be addressed to L.A.P (lapennacchio@lbl.gov), A.V. (AVisel@lbl.gov) or M.O. (marco.osterwalder@unibe.ch).

Research involving human participants, their data, or biological material

Policy information about studies with human participants or human data. See also policy information about sex, gender (identity/presentation), and sexual orientation and race, ethnicity and racism.

Reporting on sex and gender	n/a
Reporting on race, ethnicity, or other socially relevant groupings	n/a
Population characteristics	n/a
Recruitment	n/a
Ethics oversight	n/a

Note that full information on the approval of the study protocol must also be provided in the manuscript.

Field-specific reporting

Please select the one below that is the best fit for your research. If you are not sure, read the appropriate sections before making your selection.

☒ Life sciences ☐ Behavioural & social sciences ☐ Ecological, evolutionary & environmental sciences

For a reference copy of the document with all sections, see nature.com/documents/nr-reporting-summary-flat.pdf

Life sciences study design

All studies must disclose on these points even when the disclosure is negative.

Sample size	Details are provided in the Methods section including specific information related to the experimental approach used.
Data exclusions	Mouse embryos were excluded from further analysis if the developmental stage was not correct or if they were not of the genotype of interest. No data exclusions except for well-established criteria in specific data filtering steps.
Replication	Experiments were conducted in replicates when the outcome was likely to have a significant impact on the interpretation of results.<br>For transient transgenic reporter analysis in mouse embryos, sample size selection and scoring criteria were based on our experience of performing transgenic mouse assays for >3,000 total putative enhancers (VISTA Enhancer Browser: http://enhancer.lbl.gov). For enSERT, transgenic results were confirmed in at least n=2 independent biological replicates while using criteria consistent with the pipeline established for the VISTA EnhancerBrowser (see Methods).<br>For embryonic results from genetically modified mouse lines sample sizes were selected empirically based on our previous studies and the standard in the field. Phenotypic characterization of knockout mice employed a matched littermate selection strategy. The minimal number of biological replicates analyzed per experiment is indicated in the respective Methods paragraphs. Briefly, for skeletal analysis at P0, fore- and hindlimbs of at least n=2 biological replicates were analyzed for control genotypes and at least n=3 for genotypes involving the iTADd allele.<br>For ISH results at least n=3 embryos (biological replicates) were analyzed.
Randomization	Randomization was not required given the observational nature of the study and inclusion of appropriate biological replicates.
Blinding	Embryonic littermates were dissected and processed blind to genotype. Individuals who qualitatively assessed the results of in vivo transgenic reporter assays or measured skeletal elements were blinded to genotyping information.

Reporting for specific materials, systems and methods

We require information from authors about some types of materials, experimental systems and methods used in many studies. Here, indicate whether each material, system or method listed is relevant to your study. If you are not sure if a list item applies to your research, read the appropriate section before selecting a response.

Materials & experimental systems

n/a	Involved in the study	
☐	☒	Antibodies
☒	☐	Eukaryotic cell lines
☒	☐	Palaeontology and archaeology
☐	☒	Animals and other organisms
☒	☐	Clinical data
☒	☐	Dual use research of concern
☒	☐	Plants

Methods

n/a	Involved in the study	
☒	☐	ChIP-seq
☒	☐	Flow cytometry
☒	☐	MRI-based neuroimaging

Antibodies

Antibodies used	anti-digoxigenin antibody (1:5000, Roche cat. no. 11093274910)
Validation	The anti-digoxigenin Ab was validated in numerous mouse studies.

Animals and other research organisms

Policy information about studies involving animals; ARRIVE guidelines recommended for reporting animal research, and Sex and Gender in Research

Laboratory animals	Mice used for transient transgenic reporter analysis and mice of the Gli3d, mm1179d and iTADd line (strain: FVB/NJ) were housed at the LBNL Animal Care Facility, which is fully accredited by AAALAC International. Mice were maintained with water supply on a 12:12 light-dark cycle, with relative humidity set at 30–70% and a temperature of 20–26.2 °C. Mice were housed in standard micro-isolator cages on hard-wood bedding with enrichment consisting of crinkle-cut naturalistic paper strands and fed on ad libitum PicoLab Rodent Diet 20 (5053).<br>All mice were health checked and monitored daily for food and water intake by trained personnel. Euthanasia at LBNL was performed in the home cage using CO2 asphyxiation while ensuring gradual fill and displacement rate.
Wild animals	n/a
Reporting on sex	Animals of both sexes were used in these analyses. Sex was not considered as a variable in our embryonic studies since limb development is expected to show minimal differences at the respective stages of development.
Field-collected samples	n/a
Ethics oversight	All animal work at Lawrence Berkeley National Laboratory (LBNL, CA, USA) was reviewed and approved by the LBNL Animal Welfare Committee under protocol numbers 290003 and 290008.

Note that full information on the approval of the study protocol must also be provided in the manuscript.

Plants

Seed stocks	*Report on the source of all seed stocks or other plant material used. If applicable, state the seed stock centre and catalogue number. If plant specimens were collected from the field, describe the collection location, date and sampling procedures.*
Novel plant genotypes	*Describe the methods by which all novel plant genotypes were produced. This includes those generated by transgenic approaches, gene editing, chemical/radiation-based mutagenesis and hybridization. For transgenic lines, describe the transformation method, the number of independent lines analyzed and the generation upon which experiments were performed. For gene-edited lines, describe the editor used, the endogenous sequence targeted for editing, the targeting guide RNA sequence (if applicable) and how the editor was applied.*
Authentication	*Describe any authentication procedures for each seed stock used or novel genotype generated. Describe any experiments used to assess the effect of a mutation and, where applicable, how potential secondary effects (e.g. second site T-DNA insertions, mosiacism, off-target gene editing) were examined.*
